# 1689. Comparison of Antibiotics Use between Patients with Kawasaki Disease and Propensity Score-matched Controls: a Cohort Study of 106,908 Patients

**DOI:** 10.1093/ofid/ofad500.1522

**Published:** 2023-11-27

**Authors:** Tae-Hwan Kim, Ji Seong Shin, Sin Young Kim, Min Sik Jang, Jihye Kim

**Affiliations:** Hallym University College of Medicine, Anyang City, Gyeonggi-Do, Kyonggi-do, Republic of Korea; Kangdong Sacred Heart Hospital, Gangdong-gu, Seoul-t'ukpyolsi, Republic of Korea; Kangdong Sacred Heart Hospital, Gangdong-gu, Seoul-t'ukpyolsi, Republic of Korea; Kangdong Sacred Heart Hospital, Gangdong-gu, Seoul-t'ukpyolsi, Republic of Korea; Hallym University Kangdong Sacred Heart Hospital, Gangdong-gu, Seoul-t'ukpyolsi, Republic of Korea

## Abstract

**Background:**

The etiology of the Kawasaki disease (KD) has been explained by the influence of infectious agents that interfere with the immune function in genetically susceptible children. In addition of this infectious etiology, we hypothesized that microbial imbalance in the gut from antibiotics use may be an important etiologic factor. We aimed to assess the association between antibiotics use and the development of KD.

**Methods:**

A population-based, case-control study was performed using data from 2010 to 2019 from the Health Insurance Review and Assessment Service (HIRA) database. Various profiles regarding prior antibiotics use since birth were compared between patients with KD and matched controls. Children aged < 5 years who were diagnosed with initial episode of KD from 2016 to 2019 were identified. Propensity score-matched controls without KD were selected from the general population in a 1:5 ratio. Four separate study cohorts according to four different periods of antibiotics use were created: 1) within 28 days after birth; 2) within 12 months after birth; 3) within 6 months from the index date; and 4) within 12 months from the index date. The occurrence of KD were compared between KD cases and controls.

**Results:**

Overall, 17,818 patients with KD and 89,090 matched controls were included in the analysis. Use of antibiotics within 6 months (odds ratio [OR], 1.18; 95% confidence interval [CI], 1.12–1.26) and 12 months (OR, 1.23; 95% CI, 1.14–1.32) from the index date were associated with the development of KD. The association between antibiotics use and KD (OR, 1.26; 95% CI, 1.17–1.37) was most prominent in patients who had received more than three classes of antibiotics within 12 months from the index date. No significant association was observed between the duration of antibiotics use, use of all three major antibiotics classes, or use of any specific antibiotics class and KD.

**Conclusion:**

The development of KD is associated with antibiotics use. However, evidence suggesting that antibiotics use in the neonatal period or within the first 12 months of life is associated with KD is weak.

**Disclosures:**

**All Authors**: No reported disclosures

